# Prevalence and temporal trends of prostate diseases among inpatients with cardiovascular disease: a nationwide real-world database survey in Japan

**DOI:** 10.3389/fcvm.2023.1236144

**Published:** 2023-10-19

**Authors:** Kohei Kaneta, Atsushi Tanaka, Michikazu Nakai, Yoko Sumita, Hidehiro Kaneko, Mitsuru Noguchi, Koichi Node

**Affiliations:** ^1^ Department of Cardiovascular Medicine, Saga University, Saga, Japan; ^2^ Department of Medical and Health Information Management, National Cerebral and Cardiovascular Center, Suita, Osaka, Japan; ^3^ The Department of Cardiovascular Medicine, The University of Tokyo, Tokyo, Japan; ^4^ The Department of Advanced Cardiology, The University of Tokyo, Tokyo, Japan; ^5^ Department of Urology, Saga University, Saga, Japan

**Keywords:** prostate disease, benign prostate hyperplasia, prostate cancer, cardiovascular disease, acute coronary syndrome, heart failure, epidemiology, temporal trend

## Abstract

**Introduction:**

Benign prostate hyperplasia (BPH) and prostate cancer (PCa) are major prostate diseases that potentially share cardiometabolic risk factors and an elevated risk for cardiovascular disease (CVD). However, the prevalence of prostate diseases among patients with established CVD remains unclear.

**Materials and methods:**

This nationwide retrospective study assessed the prevalence and temporal trend of prostate diseases (i.e., BPH or PCa) among patients hospitalized for CVDs in Japan. We used a claims database (the Japanese Registry of All Cardiac and Vascular Diseases–Diagnosis Procedure Combination), which included data on 6,078,487 male patients recorded from 1,058 hospitals between April 2012 and March 2020. We conducted the Cochran–Armitage trend test and calculated the adjusted odds ratio (aOR) with 95% confidence intervals (CIs).

**Results:**

The prevalence of prostate diseases over the entire study period was 5.7% (BPH, 4.4%; PCa, 1.6%). When dividing the overall cohort into age categories (<65, 65–74, and ≥75 years old), the prevalence was 1.1%, 4.7%, and 9.9%, respectively (*P* for trend <0.05). In addition, the annual prevalence showed a modest increasing trend over time. Patients admitted for heart failure (HF) were significantly associated with a higher incidence of coexisting prostate diseases than those admitted for non-HF causes [aOR 1.02 (95% CI, 1.01–1.03)] or acute coronary syndrome [aOR 1.19 (95% CI, 1.17–1.22)].

**Conclusions:**

The nationwide real-world database revealed that the prevalence of prostate diseases is increasing among patients hospitalized for CVD, particularly HF. Attention to detailed causality and continued surveillance are needed to further clarify the clinical characteristics of prostate diseases among patients with CVD.

## Introduction

Benign prostate hyperplasia (BPH) and prostate cancer (PCa) are major prostate diseases among older men, and the global burden of these diseases continues to increase in the current aging and long-lived society (1–5). BPH is the most common cause of male lower urinary tract symptoms (LUTS) (6), adversely affecting the quality of life and cardiovascular outcomes (7). Patients with PCa also have an increased risk of developing cardiovascular diseases (CVDs) (8), which are a major cause of noncancer death among PCa survivors (9, 10).

The incidence of major CVDs, such as coronary heart disease and heart failure (HF), generally increases as age, dysregulated cardiometabolic risk factors, and inflammation increase (11–13). Furthermore, metabolic syndrome and coexisting proinflammatory status play a pivotal role in the pathogenesis of major prostate diseases, including BPH and PCa, and disrupted cardiometabolic conditions are strongly associated with an increased prevalence of these prostate diseases (14–18). Hence, prostate diseases share foundational risk factors with CVD and can represent an aspect of cardiometabolic syndrome (19). Therefore, a biological rationale for close interplay exists between such prostate diseases and CVD entities.

Numerous epidemiological and observational studies have shown an increased risk for CVD among patients with major prostate diseases (7, 8). This finding is helpful, especially for urologists (rather than cardiologists), allowing them to recognize that male patients with prostate diseases have a substantial risk for CVD and require a careful cardiovascular risk assessment in the urological care setting (20, 21). However, real-world clinical reports on the prevalence of prostate diseases among patients with entire or specific CVD are limited. Although prostate diseases are closely linked to CVD, knowledge and evidence concerning the burden of prostate diseases in the cardiovascular care setting are still insufficient.

The present study therefore assessed the burden of major prostate diseases and recent associated temporal trends among hospitalized patients with established CVD using a nationwide real-world database collected throughout Japan.

## Materials and methods

### Ethics

This study was approved by the Japanese Circulation Society (JCS) (No. 2020-08) and the Ethics Committee of Saga University Hospital (No. 2021-08-01 on November 01, 2021) and conducted according to the principles of the Declaration of Helsinki. The database used in this study deidentified personal information; therefore, informed consent from our study participants was not needed.

### Data sources and availability

This retrospective study obtained data from the Japanese Registry of All Cardiac and Vascular Diseases–Diagnosis Procedure Combination (JROAD-DPC), a nationwide claims database developed by the JCS. Details in the database were previously described elsewhere (22–25). In brief, the JROAD-DPC database, which contains data from cardiovascular training facilities certified by the JCS, includes inpatients' clinical information, such as their age, sex, diagnosis, comorbidity, hospitalization duration, and discharge status. Patients' diagnoses and comorbidities were coded using the International Classification of Disease and Related Health Problems 10th version (ICD-10) codes.

Given that the JROAD-DPC dataset is owned by the JCS for the purpose of research, the data that support our findings will not be shared without JCS' permission. The dataset will be available upon reasonable request from researchers and after approval by the JCS. Inquiries are to be addressed to the JCS (contact via itdatabase@j-circ.or.jp).

### Diagnoses, measurements, and definitions

In the JROAD-DPC database, each diagnosis is coded for the main diagnosis, admission-precipitating diagnosis, most resource-consuming diagnosis, and second-most resource-consuming diagnosis. A maximum of 10 diagnoses are coded for comorbidities encountered during admission and conditions that arise after admission.

We defined prostate diseases as BPH and PCa, coded as N40 and C61 in the ICD-10 codes, respectively, and identified them in any of the following data categories: the main diagnosis, admission-precipitating diagnosis, most resource-consuming diagnosis, second-most resource-consuming diagnosis, comorbidities during admission, and conditions arising after admission. Complications (hypertension, diabetes, dyslipidemia, and atrial fibrillation/flutter) were identified using the Charlson comorbidity index. Furthermore, two cardiovascular causes [acute coronary syndrome (ACS) and HF] of the index hospitalization were extracted from data categories, including the main diagnosis, admission-precipitating diagnosis, and most resource-consuming diagnosis, using the ICD-10 codes I200 (unstable angina pectoris) and I21 (acute myocardial infarction) for ACS and I50 for HF. We also extracted data on the age, sex, height, weight, body mass index (BMI), and smoking status.

### Study population

**Figure 1:**
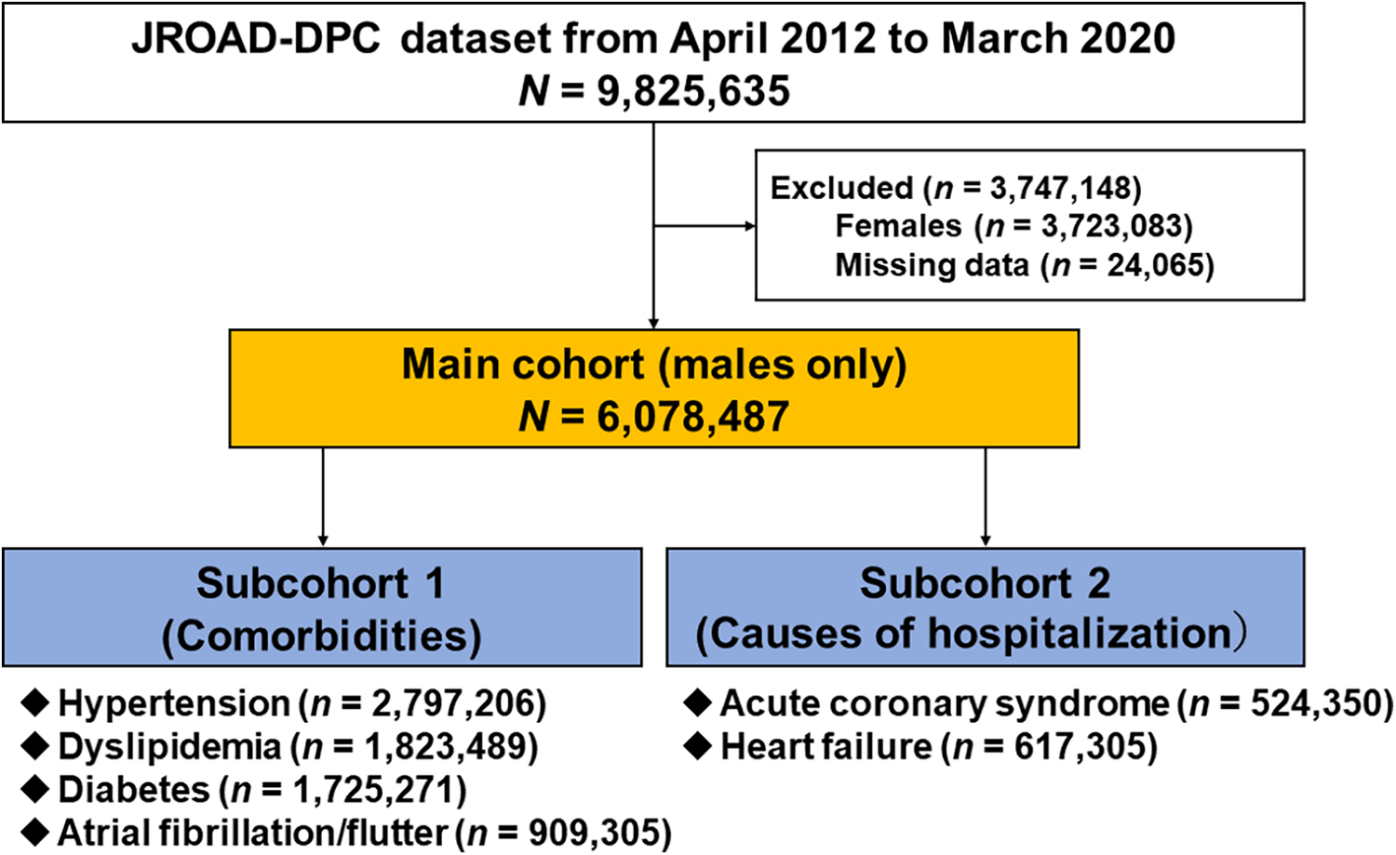
Study flowchart. JROAD-DPC, Japanese registry of all cardiac and vascular diseases–diagnosis procedure combination.

Of the 9,825,635 patients hospitalized for any CVDs recorded from 1,058 JCS-certified hospitals between April 2012 and March 2020 (Figure 1), 6,078,487 men without missing clinical data were included in the main analysis (main cohort). Next, we extracted six specific subgroups according to comorbidities (hypertension, diabetes, dyslipidemia, and atrial fibrillation/flutter) (subcohort 1) and cardiovascular causes of admission (ACS and HF) (subcohort 2). In addition, the cohorts were subdivided into 3 age groups: <65, 65–74, and ≥75 years old.

### Statistical analyses

We presented categorical data as numbers (percentages) and continuous data as medians [interquartile ranges (IQRs)]. Annual frequencies of prostate diseases were calculated according to the data obtained from April of the corresponding year to March of the following year. Using the Cochran–Armitage trend test, we assessed the prevalence of prostate diseases by age group (<65, 65–74, and ≥75 years old). To determine the odds ratios and 95% confidence intervals, we constructed multilevel mixed-effects logistic regression with institution as a random intercept and adjusting for confounding factors, such as age, smoking status, hypertension, dyslipidemia, diabetes, and atrial fibrillation/flutter. In the subcohort analyses, patients with both ACS and HF were excluded in order to analyze the associations between each cardiovascular cause of admission (ACS and HF) and prostate disease individually.

All statistical data were analyzed using the STATA16 software program (College Station, TX, USA).

## Results

### Clinical characteristics

Of the 9,825,635 patients registered in the JROAD-DPC database between April 2012 and March 2020, 2,723,083 women and 24,065 cases with missing data were excluded (Figure 1). Ultimately, 6,078,487 male patients were analyzed as the main cohort. Overall, the median age was 71 (IQR: 63, 79) years old, and the median BMI was 23.2 (IQR: 20.8, 25.7) kg/m^2^. The prevalences of current or past smoking and coexisting hypertension, dyslipidemia, diabetes, and atrial fibrillation/flutter were 56.5%, 46.0%, 30.0%, 28.4%, and 15.0%, respectively (Table 1).

**Table 1:** Clinical characteristics of the main cohort.

	Overall	Without prostate disease	With prostate disease		
			BPH and/or PCa	BPH	PCa
Number of patients	6,078,487	5,729,194	349,293	268,778	96,131
Age, years	71 (63, 79)	71 (62, 79)	79 (73, 84)	79 (73, 84)	79 (73, 84)
Height, cm	164.0 (159.0, 169.0)	164.0 (159.0, 169.0)	162.0 (158.0, 167.0)	162.0 (158.0, 167.0)	162.0 (157.0, 167.0)
Weight, kg	62.0 (53.7, 70.2)	62.0 (53.8, 70.5)	60.0 (52.6, 67.1)	60.0 (52.5, 67.1)	60.0 (53.0, 67.4)
Body mass index, kg/m^2^	23.2 (20.8, 25.7)	23.2 (20.8, 25.7)	22.9 (20.6, 25.1)	22.8 (20.6, 25.1)	23.0 (20.8, 25.2)
Smoking (current or past)	2,969,835 (56.5)	2,810,891 (56.8)	158,944 (51.9)	123,429 (52.4)	42,442 (50.4)
Hypertension	2,797,206 (46.0)	2,608,896 (45.5)	188,310 (53.9)	154,391 (57.4)	40,672 (42.3)
Dyslipidemia	1,823,489 (30.0)	1,726,845 (30.1)	96,644 (27.7)	79,213 (29.5)	20,630 (21.5)
Diabetes	1,725,271 (28.4)	1,629,641 (28.4)	95,630 (27.4)	75,821 (28.2)	23,311 (24.2)
Atrial fibrillation/flutter	909,305 (15.0)	847,553 (14.8)	61,752 (17.7)	49,959 (18.6)	13,965 (15.5)

Data are presented as the median (interquartile range) or numbers (percentages).

BPH, benign prostate hyperplasia; PCa, prostate cancer.

### Prevalence of prostate diseases

In the main cohort, 349,293 (5.7%) patients had prostate diseases (BPH: 268,778 [4.4%]; PCa: 96,131 [1.6%]), and they tended to be older and smoked less than those without such diseases. In addition, hypertension and atrial fibrillation/flutter were more common in those with prostate diseases than in those without such diseases, especially those with BPH (Table 1).

**Figure 2:**
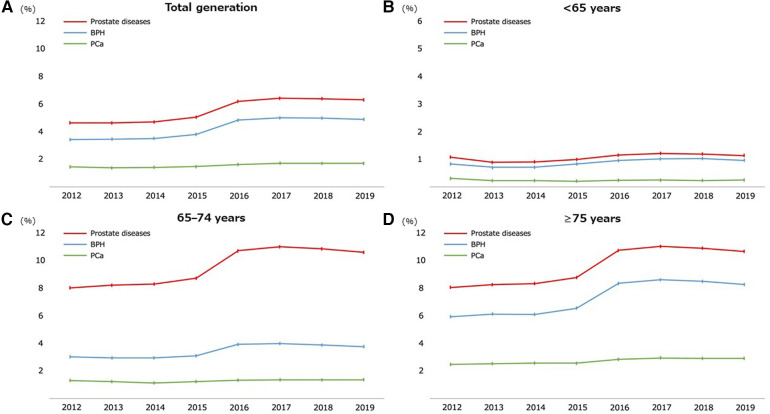
Temporal trend of the prevalence of prostate diseases from 2012 to 2019. Participants throughout the total generation (**A**) and stratified by age groups of <65 (**B**), 65–74 (**C**), and ≥75 years old (**D**). BPH, benign prostate hyperplasia; PCa, prostate cancer.

Regarding age, the prevalence of prostate diseases and individual components (BPH or PCa) significantly increased as the age increased (all *P*-values for trend <0.05) (Table 2). Figure 2 shows the annual prevalence of prostate diseases and individual components in the overall cohort and each age category from 2012 to 2019. The overall prevalence modestly increased over time in all generations, especially from 2015 to 2016 and among those who were ≥65 years old.

**Table 2:** Prevalence of prostate diseases according to age and subcohorts.

	Total generation	<65 years	65–74 years	≥75 years
Main cohort	(*N* = 6,078,487)	(*N* = 1,758,139)	(*N* = 1,876,056)	(*N* = 2,444,292)
Prostate disease (BPH and/or PCa)	349,293 (5.7)	18,945 (1.1)	87,481 (4.7)	242,867 (9.9)
BPH	268,778 (4.4)	15,739 (0.9)	66,903 (3.6)	186,136 (7.7)
PCa	96,131 (1.6)	3,891 (0.2)	24,817 (1.3)	67,423 (2.8)
Subcohort (Hypertension)	(*n* = 2,797,206)	(*n* = 753,246)	(*n* = 910,993)	(*n* = 107,557)
Prostate disease (BPH and/or PCa)	188,310 (6.7)	9,891 (1.3)	46,312 (5.1)	132,107 (11.7)
BPH	154,391 (5.5)	8,806 (1.2)	38,028 (4.2)	107,557 (9.5)
PCa	40,672 (1.5)	1,338 (0.2)	9,893 (1.1)	29,441 (2.6)
Subcohort (Dyslipidemia)	(*n* = 1,823,489)	(*n* = 560,581)	(*n* = 638,118)	(*n* = 624,790)
Prostate disease (BPH and/or PCa)	96,644 (5.3)	6,309 (1.1)	28,635 (4.5)	61,700 (9.9)
BPH	79,213 (4.3)	5,576 (1.0)	23,537 (3.7)	50,100 (8.0)
PCa	20,630 (1.1)	881 (0.2)	5,985 (0.9)	13,764 (2.2)
Subcohort (Diabetes)	(*n* = 1,725,271)	(*n* = 444,247)	(*n* = 617,700)	(*n* = 663,324)
Prostate disease (BPH and/or PCa)	95,630 (5.5)	5,696 (1.3)	27,157 (4.4)	62,777 (9.5)
BPH	75,821 (4.4)	4,961 (1.1)	21,646 (3.5)	49,214 (7.4)
PCa	23,311 (1.4)	879 (0.2)	6,602 (1.1)	15,830 (2.4)
Subcohort (Atrial fibrillation/flutter)	(*n* = 909,305)	(*n* = 199,451)	(*n* = 280,399)	(*n* = 429,455)
Prostate disease (BPH and/or PCa)	61,752 (6.8)	2,151 (1.1)	12,954 (4.6)	46,647 (10.9)
BPH	49,959 (5.5)	1,839 (0.9)	10,660 (3.8)	37,460 (8.7)
PCa	13,965 (1.5)	366 (0.2)	2,750 (1.0)	10,849 (2.5)
Subcohort (ACS)	(*n* = 524,350)	(*n* = 184,470)	(*n* = 167,594)	(*n* = 172,286)
Prostate disease (BPH and/or PCa)	23,413 (4.5)	1,623 (0.9)	6,820 (4.1)	14,970 (8.7)
BPH	18,834 (3.6)	1,429 (0.8)	5,529 (3.3)	11,876 (6.9)
PCa	5,263 (1.0)	220 (0.1)	1,466 (0.9)	3,577 (2.1)
Subcohort (HF)	(*n* = 617,305)	(*n* = 105,556)	(*n* = 132,524)	(*n* = 379,225)
Prostate disease (BPH and/or PCa)	53,260 (8.6)	1,190 (1.1)	6,486 (4.9)	45,584 (12.0)
BPH	41,827 (6.8)	1,042 (1.0)	5,263 (4.0)	35,522 (9.4)
PCa	13,383 (2.2)	166 (0.2)	1,413 (1.1)	11,804 (3.1)

Data are presented as numbers (percentages).

ACS, acute coronary syndrome; BPH, benign prostate hyperplasia; HF, heart failure; PCa, prostate cancer.

### Subcohort analyses

Table 2 also lists the prevalence of prostate diseases by each subcohort. Similar to that in the main cohort, the prevalence increased significantly with age in each subcohort (all *P*-values for trend <0.05), and the time trend for the prevalence also showed a modest increase over time (Supplementary Figures S1–S6).

In the subcohort analysis, the lowest and highest prevalence rates of prostate diseases were observed in the subcohorts with ACS (4.5%) and HF (8.6%) (Table 2). The closer association of prostate diseases with HF was validated through univariate and multivariate logistic regression analyses (Table 3 and Supplementary Table S1). Overall, the subcohort admitted for HF had significantly higher incidences of prostate diseases, dominated by BPH, than the subcohorts admitted for non-HF causes or ACS (Table 3). When stratifying these subcohorts into three age categories, the association of subcohorts admitted for HF with the coexistence of prostate diseases was more pronounced in the older group (≥65 years old) (Supplementary Table S1).

**Table 3:** Univariate and multivariate logistic regression analyses of prostate disease incidences in subcohorts according to the cause of admission.

Incidence	Causes of admission	Crude		Adjusted^a^	
		Odds ratio (95% CI)	*P*-value	Odds ratio (95% CI)	*P*-value
Prostate disease (BPH and/or PCa)	HF vs. Non-HF (reference)	1.59 (1.58–1.61)	<0.001	1.02 (1.01–1.03)	0.003
	HF vs. ACS (reference)	2.01 (1.97–2.04)	<0.001	1.19 (1.17–1.22)	<0.001
BPH	HF vs. Non-HF (reference)	1.62 (1.60–1.64)	<0.001	1.03 (1.01–1.04)	<0.001
	HF vs. ACS (reference)	1.93 (1.90–1.97)	<0.001	1.17 (1.14–1.19)	<0.001
PCa	HF vs. Non-HF (reference)	1.38 (1.35–1.41)	<0.001	0.94 (0.92–0.96)	<0.001
	HF vs. ACS (reference)	2.17 (2.10–2.24)	<0.001	1.25 (1.20–1.30)	<0.001

ACS, acute coronary syndrome; BPH, benign prostate hyperplasia; CI, confidence interval; HF, heart failure; PCa, prostate cancer.

^a^ Adjusted by factors of age, smoking status, hypertension, dyslipidemia, diabetes, and atrial fibrillation/flutter.

## Discussion

This study used a nationwide claims database recorded from JCS-certified hospitals between April 2012 and March 2020. To our knowledge, this is the first study to reveal the real-world prevalence of prostate diseases among patients hospitalized for CVDs. Prostate diseases were more prevalent in older patients than younger ones and showed a modest temporal increasing trend in the past eight years (2012–2019). Furthermore, the coexistence of prostate diseases was more common in patients hospitalized for HF than in those hospitalized for other cardiovascular causes, including ACS. Our findings highlight the potential clinical importance for cardiologists and even general physicians to recognize the potential association between CVD and the risk for prostate disease.

Metabolic syndrome and relevant cardiometabolic disorders, which are fully established as key risk factors for most CVDs, have been recently reported to play an important role in the pathogenesis of major prostate diseases (BPH and PCa) (17, 19, 26–29), highlighting an increased burden of prostate diseases coexisting with CVD in actual clinical settings. Epidemiological studies have reported that CVD is prevalent among patients with LUTS (primarily caused by BPH) or PCa (7, 30–32). Furthermore, some PCa medications, including ADT (androgen deprivation therapy), have a potency that adversely affects the cardiometabolic status and increases the risk for cardiovascular complications (33–36). Chan et al. (37) recently demonstrated temporal increasing trends of recipients of ADT therapy with cardiovascular risk factors, frequently used cardiovascular and metabolic medications, and developed cardiovascular events. CVD is the leading cause of mortality in PCa survivors (9, 10). These studies have contributed to emphasizing the clinical need for cardiovascular risk assessments and appropriate risk management in this population (20, 38, 39).

Currently, studies assessing the real-world burden of prostate diseases among patients with established CVD remain limited. In Poland, Semczuk-Kaczmarek et al. (40) reported that 62 (37.3%) out of 166 patients hospitalized with CVD had moderate-to-severe LUTS according to the International Prostate Symptoms Score. In a nationwide survey using the National Inpatient Sample in the United States between 2004 and 2014 (41), PCa was the most prevalent in a specific population with cancer who underwent percutaneous coronary intervention. Relative to these studies, the strength of the present study was being the first to attempt to survey the burden of two major prostate diseases, namely BPH and PCa, in patients hospitalized with CVD in a real-world nationwide dataset. In addition, we found a modestly increasing temporal trend of prevalent prostate diseases in the cohort examined, consistent with the global trend of increased burden of prostate diseases in general populations (1–5). This increase may reflect aging and indicate the need to promote public and clinical awareness of prostate diseases (3, 42) and PCa screening with prostate-specific antigen tests (43–45). Collectively, continued surveillance is needed to further clarify the clinical characteristics of prostate diseases among patients with CVD. Details concerning casualties should also be further examined.

In the present study, prostate diseases, dominated by BPH, were more prevalent in inpatients with HF than in those with non-HF causes or ACS, highlighting a possible association between HF and prostate diseases. Although the reason for the difference is still uncertain, it may be at least caused by the epidemiological fact that the incidence of corresponding diseases reflects demographics with aging. Lusty et al. (46) recently revealed that the most common medications for BPH (5-alpha reductase inhibitor, α-blocker, and combination therapy) were associated with an increased risk for incident cardiac failure in older (median: 73 years old) patients with BPH. More recently, HF was proven to be the leading cause of CVD admission and an increasing cause of mortality among patients across cancer types, including PCa (47, 48). Thus, considering the increasing global burden of HF (49), clinicians need to recognize that HF is an emerging and prognostic complication in patients with prostate diseases.

Although not limited to prostate diseases, estimation of the risk and early screening of the coexistence of non-CVD in patients with established CVD may lead to better overall patient outcomes. With recent improvements in diagnostics and therapeutics in various fields, including cardiology and urology, the resultant increase in the number of survivors from individual diseases and aging will further augment the subsequent risk of developing cardiovascular and noncardiovascular complications. In particular, patients with cancer generally have an increased risk for CVD (50–52), and the development of a better healthcare system is urgently needed in order to provide multidisciplinary clinical management and appropriate cardiovascular care (“onco-cardiology”) to patients with cancer.

In the present study, we showed real-world evidence concerning the burden and temporal increasing trend of major prostate diseases (BPH and PCa) among patients hospitalized for CVD. Given the close epidemiological relationship based on shared risk factors between prostate diseases and CVD entities, the prevalence of prostate diseases identified in inpatients may be merely part of a larger and more complex issue and is also common in patients with chronic CVD and even in those at risk for CVD (14–18). However, conducting clinical interviews regarding the presence of LUTS and screening tests for prostate diseases in cardiovascular care settings is currently uncommon. Our findings may be clinically useful to motivate cardiologists and even general physicians to screen patients with CVD for the presence of prostate diseases and to share clinical information with urologists. Furthermore, our study may promote clinical and research collaboration among specialists (especially urologists and cardiologists), leading to the development of a novel academic field of “uro-cardiology” in the near future.

### Limitations

Our study has several limitations that need to be considered, and they were mainly based on the use of data sources obtained from medical claims. First, the present analysis was based only on the DPC data, and these data might contain certain inevitable errors, leading to the over- or underestimation of the clinical diagnosis accuracy. In particular, the JROAD-DPC data were obtained from the cardiovascular unit of several JCS-certified training facilities, and cardiologists might not be knowledgeable enough in the clinical diagnosis of prostate diseases. In addition, data were only collected from patients hospitalized for CVDs in JCS-certified hospitals; thus, selection bias is possible. We also did not compare the inpatients with CVD with healthy individuals or inpatients without CVD. Second, the DPC data system cannot share individual information among hospitals. If patients were admitted to another hospital, the previous DPC data could not be carried over, and the individuals could not be identified; thus, duplicates in the dataset are likely. Third, the DPC dataset did not include clinical information on the etiology, laboratory and imaging data, medications, or disease severity and staging of CVD and prostate diseases. Fourth, we had no information about the subsequent clinical course, rehospitalization, or mortality. Therefore, further research is needed to assess long-term outcomes in patients suffering from prostate diseases with established CVD. Finally, given that CVD and prostate diseases often vary in their prevalence by ethnicity and region (1, 3, 4), whether or not our findings from this Japanese patient population can be applied to different populations remains uncertain.

## Conclusions

The nationwide real-world dataset used in this study revealed the increasing prevalence of prostate diseases among patients hospitalized for CVDs, particularly HF. Cardiologists and even general physicians must recognize that prostate diseases and CVDs often share underlying risk factors and that the frequency of their coexistence is increasing. This insight may facilitate the early screening and diagnosis of prostate diseases and help improve healthcare management.

## Data Availability

Given that the JROAD-DPC dataset is owned by the JCS for the purpose of research, the data that support our findings will not be shared without JCS' permission. The dataset will be available upon reasonable request from researchers and after approval by the JCS. Inquiries are to be addressed to the JCS (contact via itdatabase@j-circ.or.jp).
