# Supplementary material for: Prevalence and temporal trends of prostate diseases among inpatients with cardiovascular disease: a nationwide real-world database survey in Japan
Source: Front Cardiovasc Med. 2023 Oct 19;10:1236144. doi: 10.3389/fcvm.2023.1236144 (PMC10620699; doi:10.3389/fcvm.2023.1236144)
Supplement: Supplementary file 1 [file Table1.docx]

**Table S1. Univariate and multivariate logistic regression analyses of prostate disease incidences in subcohorts according to the cause of admission, by age group**

| **Incidence** | **Causes of admission** | **Crude** | | **Adjusted*** | |
| --- | --- | --- | --- | --- | --- |
|  |  | **Odd ratio**  **(95% CI)** | ***P* value** | **Odd ratio**  **(95% CI)** | ***P* value** |
| <65 years | | | | | |
| Prostate disease (BPH and/or PCa) | HF vs. Non-HF (reference) | 1.04 (0.98–1.10) | 0.224 | 0.97 (0.90–1.03) | 0.290 |
|  | HF vs. ACS (reference) | 1.28 (1.18–1.38) | <0.001 | 1.24 (1.13–1.37) | <0.001 |
| BPH | HF vs. Non-HF (reference) | 1.10 (1.03–1.17) | 0.004 | 1.01 (0.94–1.08) | 0.870 |
|  | HF vs. ACS (reference) | 1.27 (1.17–1.38) | <0.001 | 1.25 (1.13–1.39) | <0.001 |
| PCa | HF vs. Non-HF (reference) | 0.69 (0.59–0.80) | <0.001 | 0.69 (0.58–0.82) | <0.001 |
|  | HF vs. ACS (reference) | 1.33 (1.08–1.63) | 0.006 | 1.20 (0.94–1.54) | 0.148 |
| 65–74 years | | | | | |
| Prostate disease (BPH and/or PCa) | HF vs. Non-HF (reference) | 1.05 (1.02–1.08) | <0.001 | 1.03 (0.99–1.06) | 0.059 |
|  | HF vs. ACS (reference) | 1.21 (1.17–1.26) | <0.001 | 1.16 (1.12–1.22) | <0.001 |
| BPH | HF vs. Non-HF (reference) | 1.12 (1.08–1.15) | <0.001 | 1.07 (1.04–1.10) | <0.001 |
|  | HF vs. ACS (reference) | 1.21 (1.16–1.26) | <0.001 | 1.17 (1.11–1.22) | <0.001 |
| PCa | HF vs. Non-HF (reference) | 0.80 (0.75–0.84) | <0.001 | 0.85 (0.80–0.90) | <0.001 |
|  | HF vs. ACS (reference) | 1.24 (1.15–1.34) | <0.001 | 1.14 (1.05–1.25) | 0.003 |
| ≥75 years | | | | | |
| Prostate disease (BPH and/or PCa) | HF vs. Non-HF (reference) | 1.27 (1.25–1.28) | <0.001 | 1.09 (1.08–1.11) | <0.001 |
|  | HF vs. ACS (reference) | 1.42 (1.39–1.45) | <0.001 | 1.25 (1.22–1.28) | <0.001 |
| BPH | HF vs. Non-HF (reference) | 1.29 (1.27–1.31) | <0.001 | 1.09 (1.07–1.10) | <0.001 |
|  | HF vs. ACS (reference) | 1.38 (1.35–1.41) | <0.001 | 1.21 (1.18–1.24) | <0.001 |
| PCa | HF vs. Non-HF (reference) | 1.12 (1.10–1.15) | <0.001 | 1.07 (1.05–1.09) | <0.001 |
|  | HF vs. ACS (reference) | 1.49 (1.43–1.55) | <0.001 | 1.34 (1.28–1.41) | <0.001 |

*Adjusted by factors such as age, smoking status, hypertension, dyslipidemia, diabetes, and atrial fibrillation/flutter.

*ACS, acute coronary syndrome; BPH, benign prostate hyperplasia; CI, confidence interval; HF, heart failure; PCa, prostate cancer.*

**Figure S1. Temporal trend in the prevalence of prostate diseases from 2012 to 2019 in the subcohort with hypertension**

**
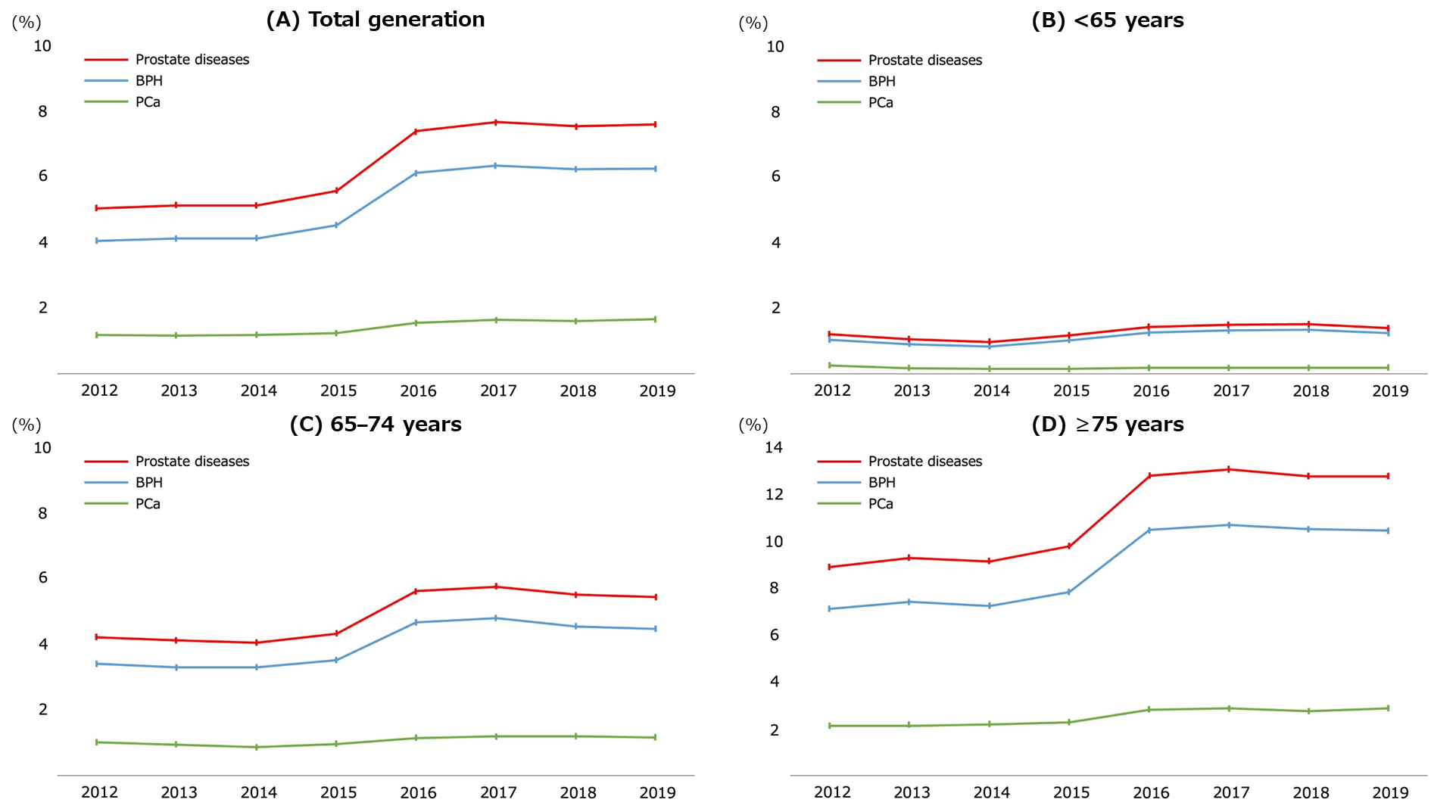
**

Participants throughout the total generation (A) and stratified by age groups of <65 (B), 65–74 (C), and ≥75 years old (D).

*BPH, benign prostate hyperplasia; PCa, prostate cancer*.

**Figure S2. Temporal trend in the prevalence of prostate diseases from 2012 to 2019 in the subcohort with dyslipidemia**

**
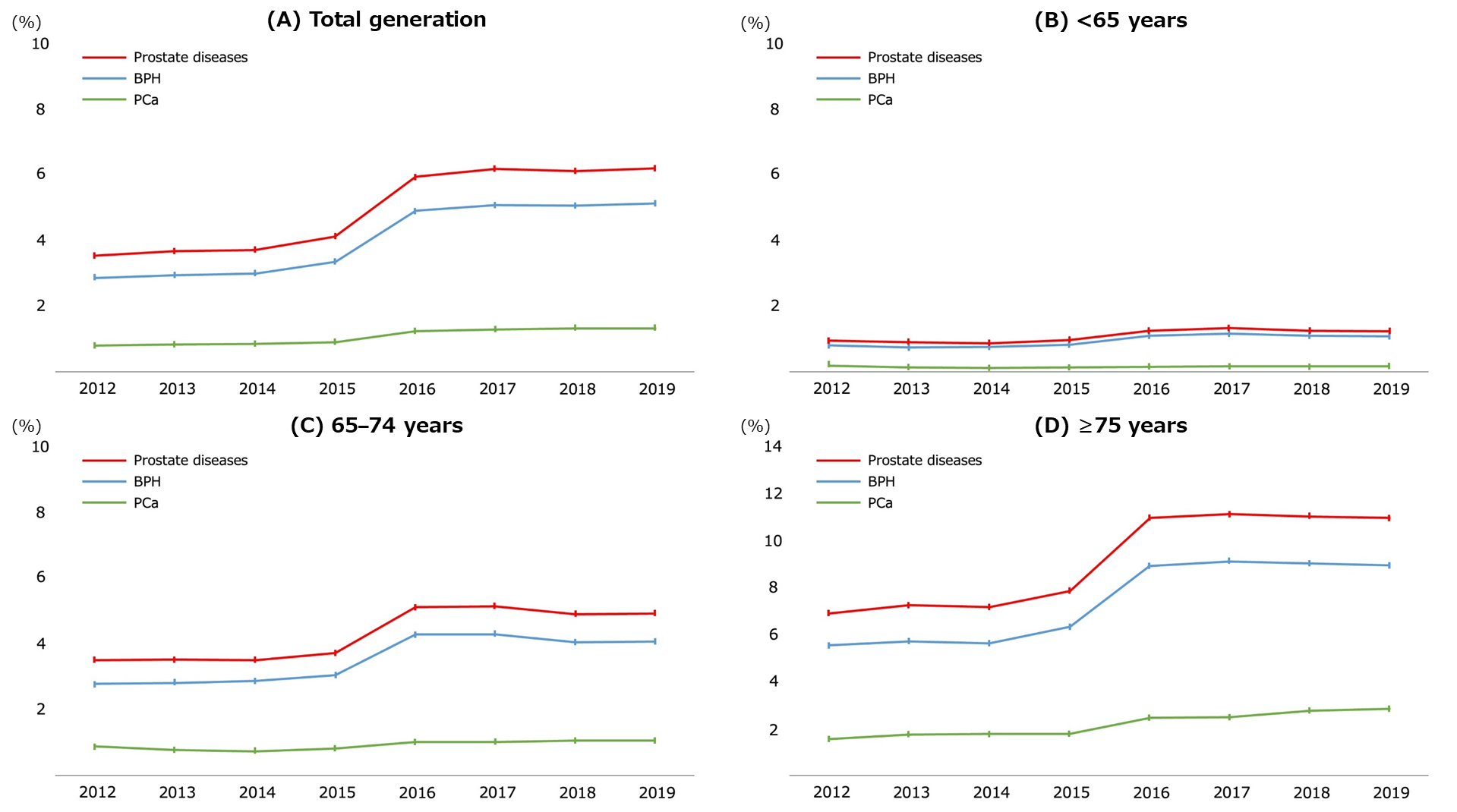
**

Participants throughout the total generation (A) and stratified by age groups of <65 (B), 65–74 (C), and ≥75 years old (D).

*BPH, benign prostate hyperplasia; PCa, prostate cancer*.

**Figure S3. Temporal trend in the prevalence of prostate diseases from 2012 to 2019 in the subcohort with diabetes**

**
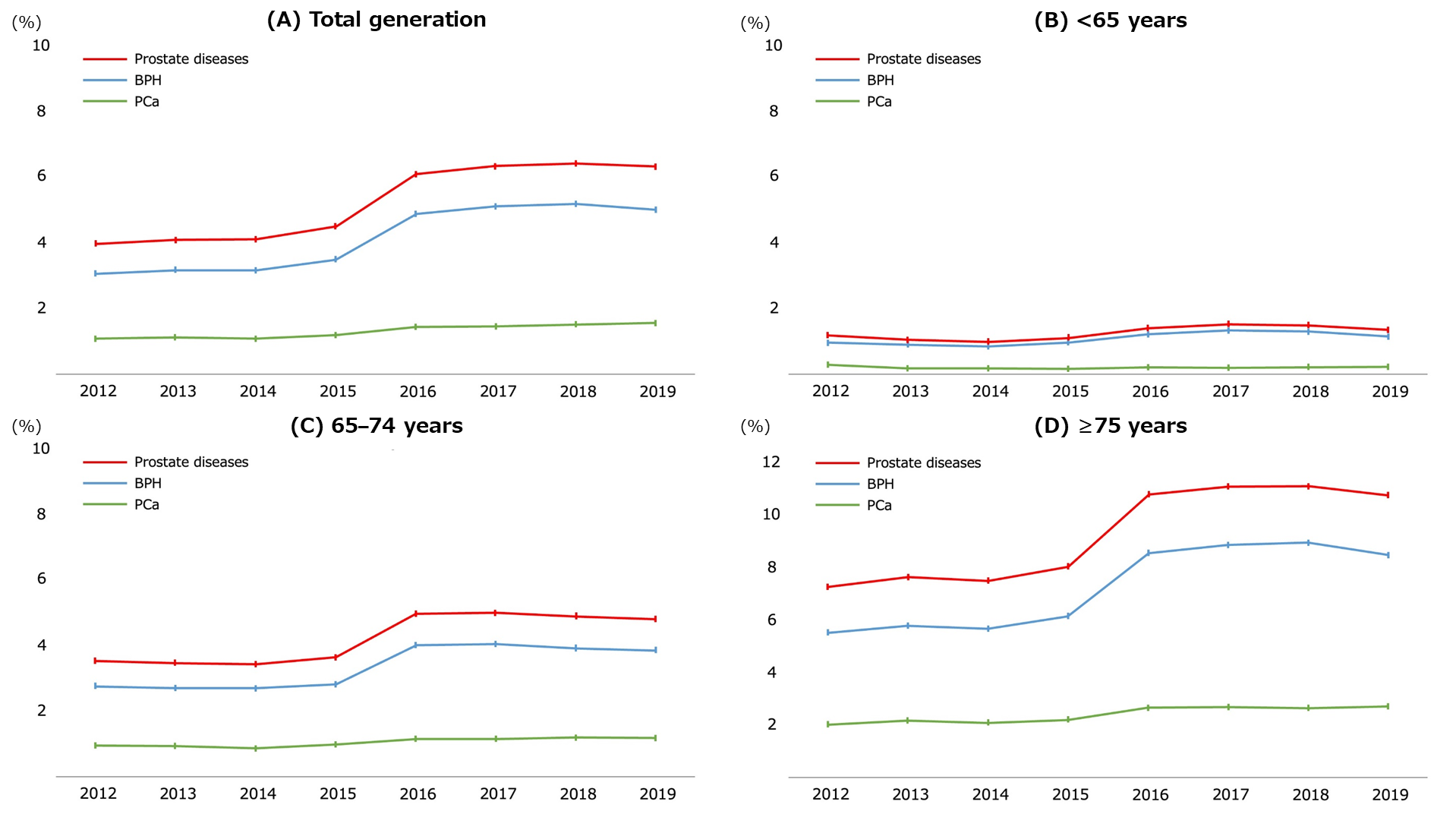
**

Participants throughout the total generation (A) and stratified by age groups of <65 (B), 65–74 (C), and ≥75 years old (D).

*BPH, benign prostate hyperplasia; PCa, prostate cancer*.

**Figure S4. Temporal trend in the prevalence of prostate diseases from 2012 to 2019 in the subcohort with atrial fibrillation/flutter**

**
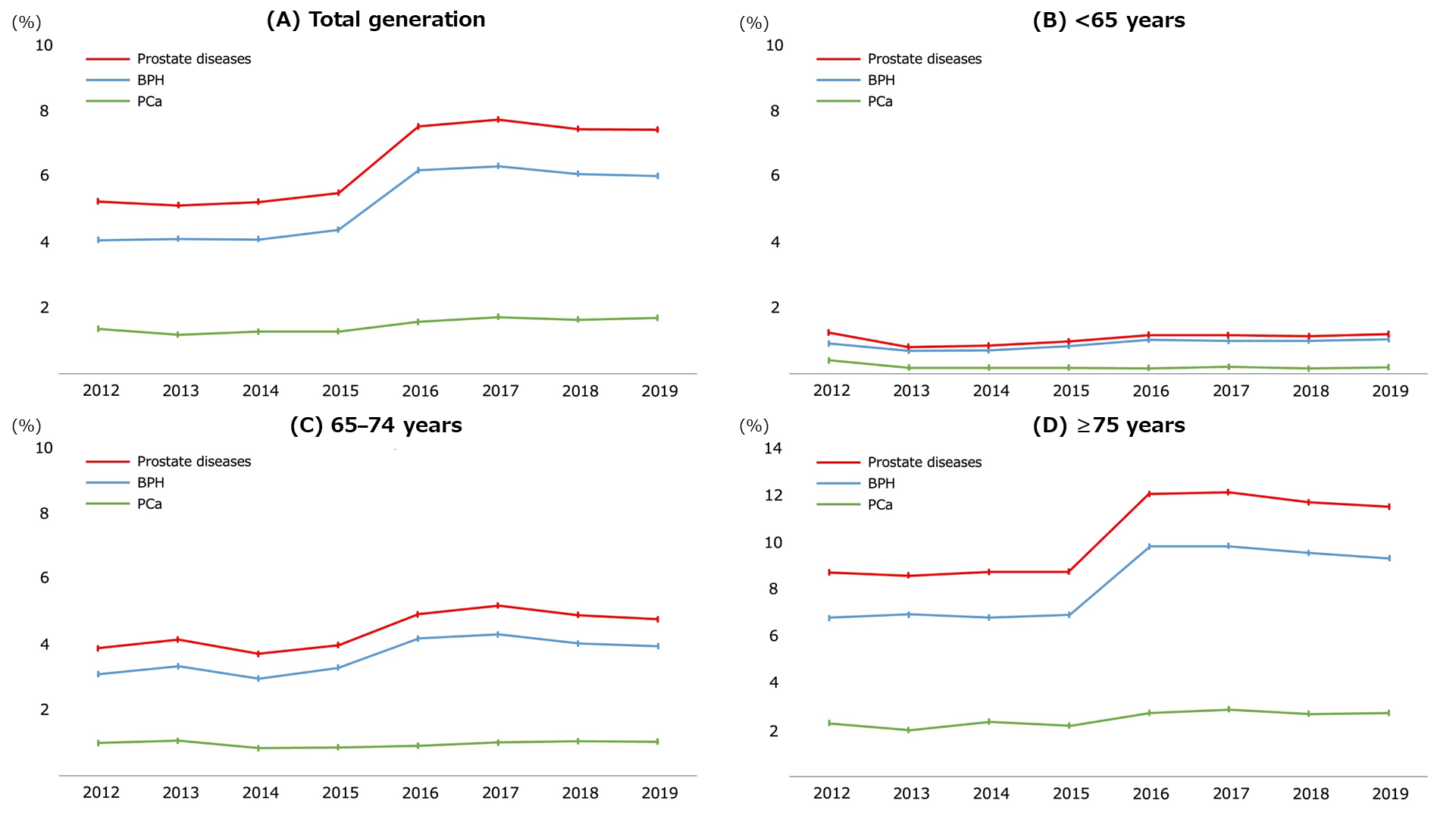
**

Participants throughout the total generation (A) and stratified by age groups of <65 (B), 65–74 (C), and ≥75 years old (D).

*BPH, benign prostate hyperplasia; PCa, prostate cancer*.

**Figure S5. Temporal trend of prevalence of prostate diseases from 2012 to 2019 in the subcohort with ACS**

**
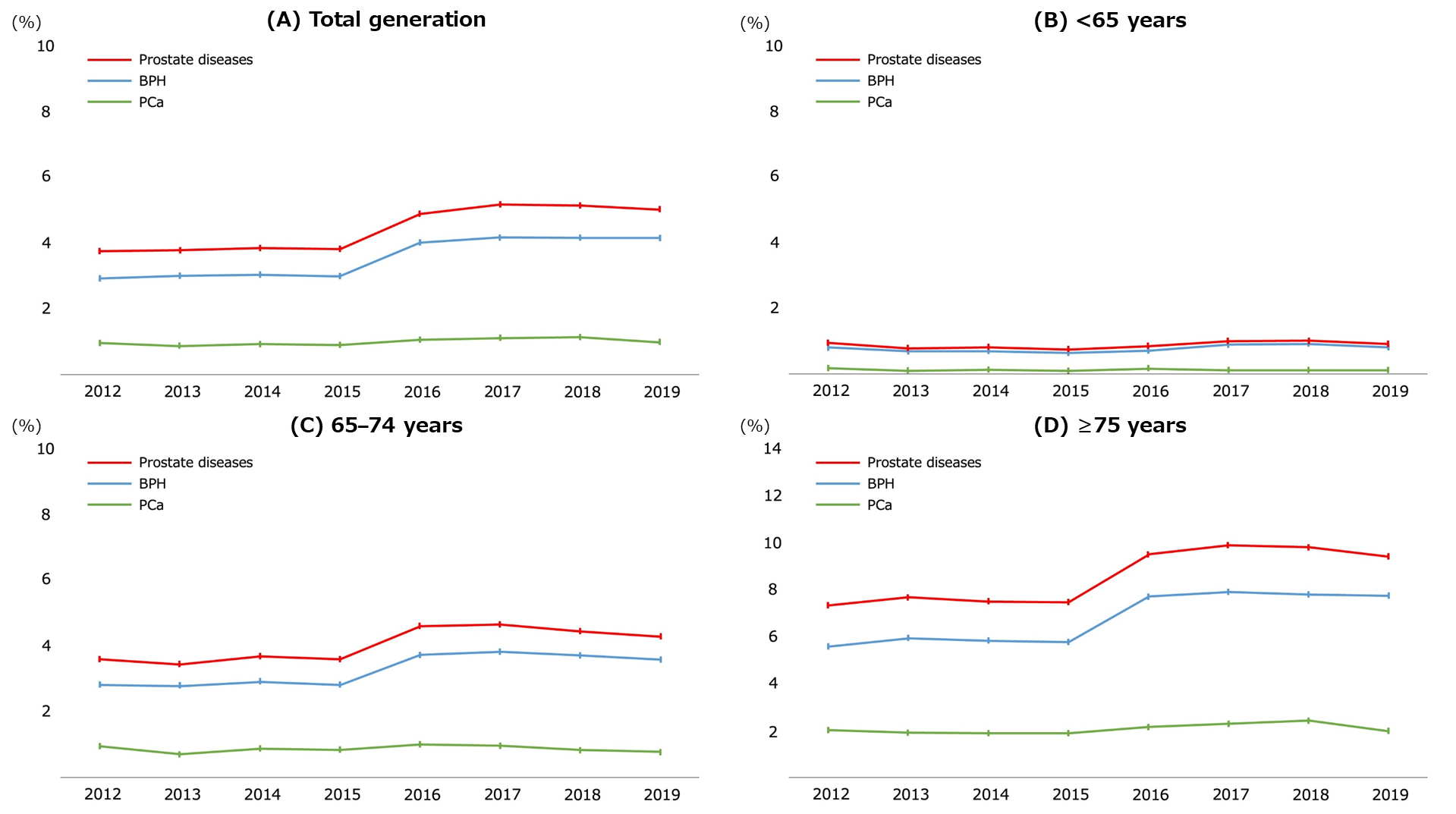
**

Participants throughout the total generation (A) and stratified by age groups of <65 (B), 65–74 (C), and ≥75 years old (D).

*ACS, acute coronary syndrome; BPH, benign prostate hyperplasia; PCa, prostate cancer*.

**Figure S6. Temporal trend of prevalence of prostate diseases from 2012 to 2019 in the subcohort with HF**

**
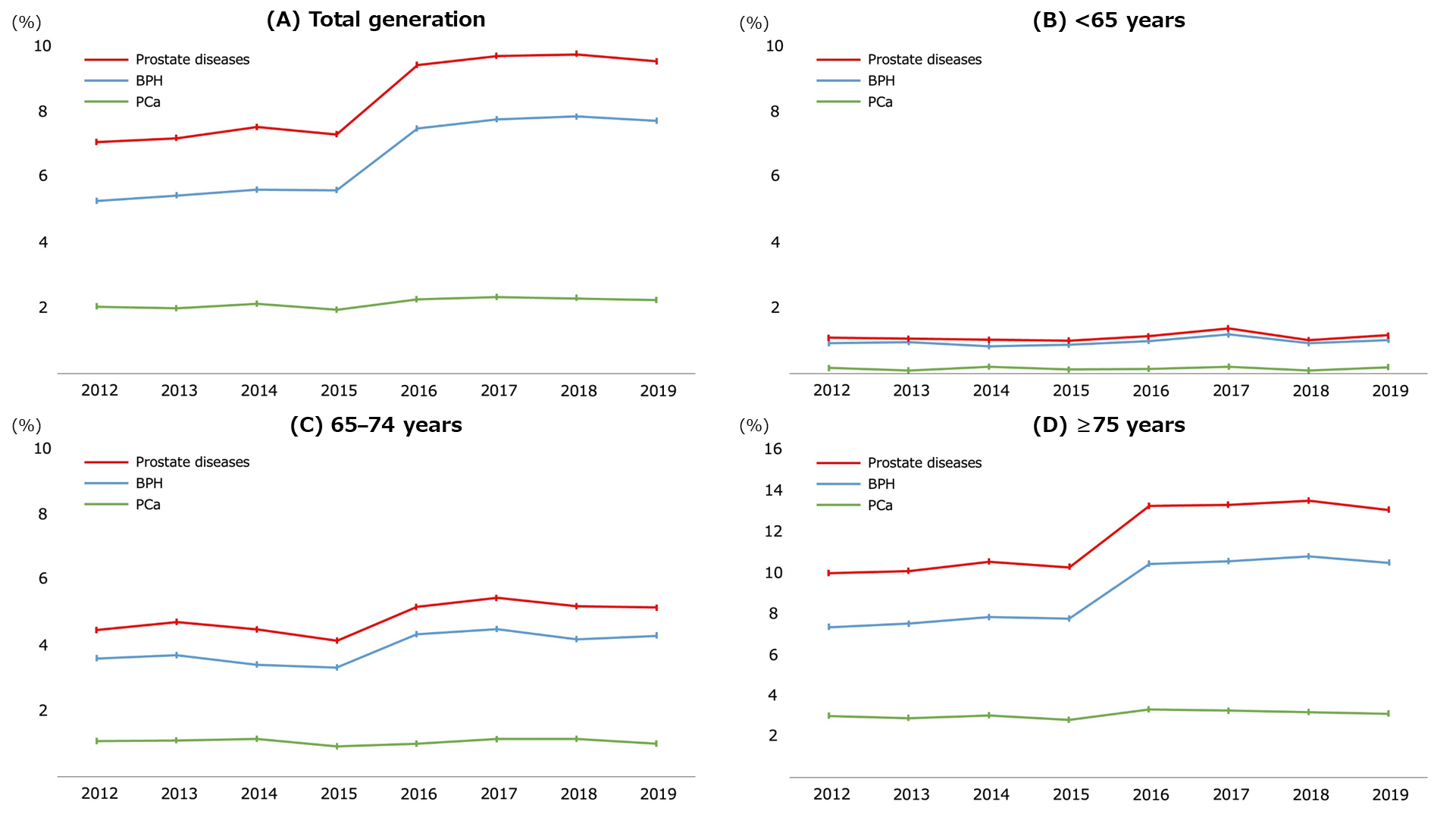
**

Participants throughout the total generation (A) and stratified by age groups of <65 (B), 65–74 (C), and ≥75 years old (D).

*BPH, benign prostate hyperplasia; HF, heart failure; PCa, prostate cancer*.
